# Small bowel mesenteric cystic lymphangioma in an adult patient: a rare case report and review of literature

**DOI:** 10.1097/RC9.0000000000000066

**Published:** 2026-01-09

**Authors:** Temesgen Agegnehu Abebe, Ashenafi Amsalu Feleke, Aklilu Yiheyis Abereha, Ermias Teklehaimanot Yefter, Endalew Demoz Worku, Weynishet Kebede Seyfu

**Affiliations:** a Department of Surgery, Debre Markos University, Debre Markos, Ethiopia; b Department of Surgery, University of Gondar, Gondar, Ethiopia; c Department of Pathology, University of Gondar, Gondar, Ethiopia; d Department of Radiology, University of Gondar, Gondar, Ethiopia

**Keywords:** abdominal mass, case report, lymphangioma, mesenteric cyst, resection and anastomosis

## Abstract

**Introduction and Importance:**

Mesenteric cystic lymphangiomas are rare intraperitoneal benign tumors of the mesentery. They are thought to arise from lymphatic vessel malformation of unknown etiology during development. They are extremely rare in adults. They occur very rarely in the peritoneum. Preoperative diagnosis is challenging. Radiologic investigations are important, but histopathology is required for definitive diagnosis. Complete surgical excision is the standard treatment.

**Case Presentation:**

A 30-year-old male patient presented with mild abdominal pain of 10 years’ duration. He appeared chronically unwell. All vital signs were in the normal range. There was a palpable abdominal mass in the right lower quadrant. CT scan suggested mesenteric cystic lymphangioma, which was confirmed by histopathology. Laparotomy with *en bloc* excision of the mass and ileoileal end-to-end anastomosis was performed. The patient was discharged on the 10th postoperative day and resumed normal activity.

**Clinical Discussion:**

Mesenteric cystic lymphangioma in adults is exceedingly rare and often presents with vague, nonspecific abdominal symptoms, making preoperative diagnosis difficult. Radiologic imaging, particularly CT scan, plays an important role in the diagnosis, but histopathologic examination remains the gold standard. Complete surgical excision with negative margins is the treatment of choice to prevent recurrence and potential complications such as obstruction, perforation, or infection.

**Conclusion:**

Mesenteric cystic lymphangioma should be considered in adults presenting with chronic abdominal mass or discomfort. Management options include aspiration and injection of sclerosant agents, radio-frequency ablation, and targeted immune-therapy. However, surgical excision remains the standard curative treatment, preferably via an open approach.

## Introduction

Lymphangiomas are cystic benign tumors arising from developmental abnormalities of the lymphatic system^[1]^. Mesenteric cystic lymphangiomas commonly occur during childhood with male predominance and account for 1:20 000 admissions. Sixty-five percent of cases are present at birth, and 90% manifest before age 2^[1–3]^. Mesenteric lymphangiomas in adults are extremely rare^[1,4]^.

The etiology of mesenteric cystic lymphangioma is unclear but is considered primarily congenital^[4]^. During embryogenesis, failure of lymphatic channels to connect with the venous system results in the development of lymphatic buds, which eventually form multicystic masses^[1]^. Acquired forms have been reported following hemorrhage or inflammatory processes involving lymphatic vessels, leading to cystic changes^[2]^. Ninety-five percent of lymphangiomas are found in the head, neck and axillary regions. Isolated small bowel lesions occur in <1% of cases but account for 70% of intra-abdominal lymphangiomas^[3]^.

**HIGHLIGHTS**

- Rare mesenteric cystic lymphangioma presenting in an adult patient.
- Preoperative diagnosis is challenging due to nonspecific symptoms.
- CT imaging aids diagnosis, but histopathology confirms the lesion.
- Complete surgical excision is the curative treatment of choice.
- Early recognition prevents complications and recurrence.

Clinical presentation ranges from asymptomatic incidental findings to emergency presentations with acute abdomen^[2]^. Diagnosis is suggested by radiologic investigation, but definitive diagnosis is by histopathology^[4]^.

Here, we present a case of mesenteric cystic lymphangioma in a 30-year-old male and its subsequent clinical management.

This case report has been reported in line with the SCARE criteria^[5]^.

## Case presentation

A 30-year-old male patient presented with vague, dull, periumblical abdominal pain of 10 years’ duration. There was no history of vomiting, abdominal distension, or change in bowel habits. He has no history of previous surgery or trauma. The patient had no prior surgery, trauma, psychiatric illness, diabetes, hypertension, or other chronic illness.

On admission, he appeared chronically unwell. Vital signs were within normal limits (BP = 100/70 mmHg, PR = 64 beats/min, RR = 20/min, T° = 36.9°C, SpO_2_ = 91% on room air). He had pink conjunctiva and non-icteric sclera. The abdomen was flat, symmetrical, and moved with respiration. There was a palpable abdominal mass in the right lower quadrant, which measures about 10 × 6 cm, non-tender, firm, mobile, not attached with the overlying structure and with a smooth surface. Physical examination was otherwise unremarkable.

**Figure 1.**
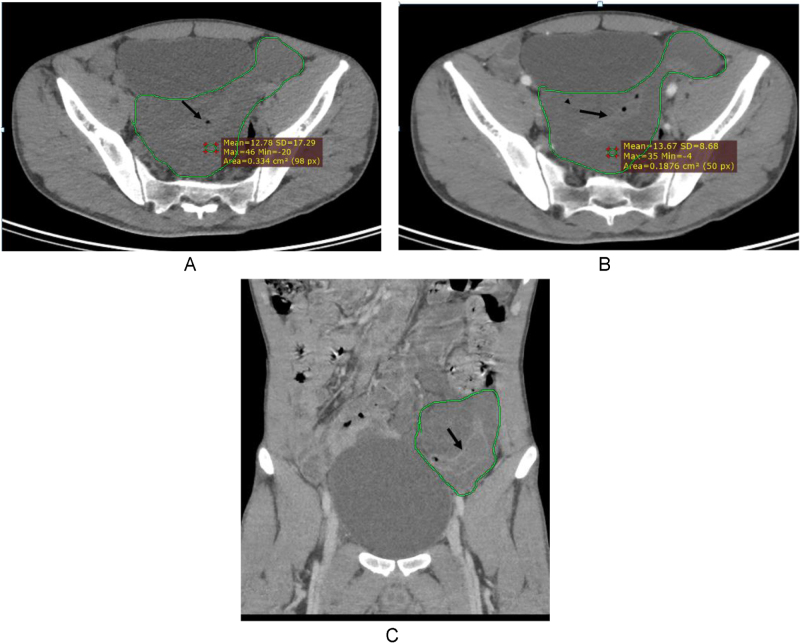
Contrast-enhanced abdominal CT scan showing a large, well-defined hypodense multiloculated cystic lesion in the small bowel mesentery. (A) Pre contrast axial CT, shows a hypodense mass in the retrovesical and paravesical region through which the bowel traverses (black arrow – traversing bowel, green ring – the mass). (B) Post-contrast axial scan, the mass has no post-contrast enhancement, but the traversing bowel has wall enhancement (black arrow – traversing bowel, green ring – the mass). (C) Post-contrast coronal image, the mass has no enhancement on post-contrast study, but the traversing bowel has wall enhancement (black arrow – traversing bowel, green ring – the mass).

Laboratory investigations showed WBC 4.34 × 10^3^/mm^3^ with lymphocyte predominance (48%), platelet count 279 × 10^3^/mm^3^, hematocrit 42%, and RBS 71.56 mg/dl. Other Complete blood cell profiles, liver function tests and renal function tests were otherwise normal (Table 1). Blood group was O positive. Abdominal CT with contrast showed an intraperitoneal cystic mass with internal traversing bowel without luminal narrowing secondary to mesenteric cystic lymphangioma. There was a 12.7 × 10.3 × 8.7 cm hypodense fluid density mass located in the retrovesical region, which extends to the left lower quadrant (Fig. 1A). There was traversing small bowel within the mass without luminal narrowing. The mass has no post-contrast enhancement (Fig. 1B and C). The small bowel and large bowel had normal wall thickness, with no sign of obstruction, dilation, or inflammation. The appendix is visualized and appears normal. There is no evidence of retroperitoneal, para-aortic, or pelvic lymphadenopathy. The CT finding of the rest of the viscera is unremarkable.

**Table 1:** Preoperative laboratory investigations

Test	Result	Normal values
Complete blood cell (CBC) count		
White blood cell	4.34 × 10^3^/mm^3^	4.0–11.0 × 10^3^/mm^3^
Lymphocyte	48%	20–40%
Granulocyte	40%	50–70%
Eosinophil	3.3%	1–6%
Monocyte	6%	2–8%
Basophil	2.7%	0–1%
Red blood cell	5.01 × 10^6^/mm^3^	4.5–5.9 × 10^6^/mm^3^ (male)
Hemoglobin	15.7 g/dl	13.5–17.5 g/dl (male)
Hematocrit	42%	41–53% (male)
Mean corpuscular volume	83.8 fl	80–100 fl
Mean corpuscular hemoglobin	31.3 pg	27–33 pg
Mean corpuscular hemoglobin concentration	37.3 g/l	32–36 g/l
Red blood cell distribution width	13.1%	11.5–14.5%
Platelet count	279 × 10^3^/mm^3^	150–450 × 10^3^/mm^3^
Mean platelet volume	10.2 fl	7.5–11.5 fl
Liver and renal function tests		
Aspartate transaminase (SGOT)	40 U/l	10–40 U/l
Alanine transaminase (SGPT)	33 U/l	7–56 U/l
Serum albumin	3.8 g/dl	3.5–5.0 g/dl
Bilirubin, direct	0.24 mg/dl	0–0.3 mg/dl
Bilirubin, total	0.9 mg/dl	0.3–1.2 mg/dl
Serum creatinine	0.77 mg/dl	0.6–1.3 mg/dl
Blood urea nitrogen	18 mg/dl	7–20 mg/dl
Other blood tests		
Random blood sugar	71.56 mg/dl	70–140 mg/dl

**Figure 2.**
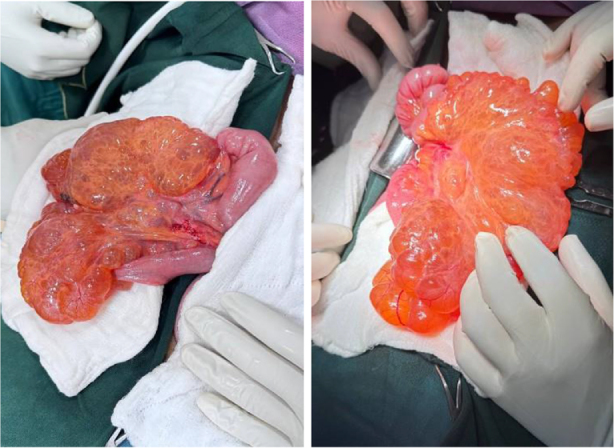
Intraoperative images showing a smooth, cystic lesion arising from the distal ileal mesentery encroaching on approximately 20 cm of bowel segment.

**Figure 3.**
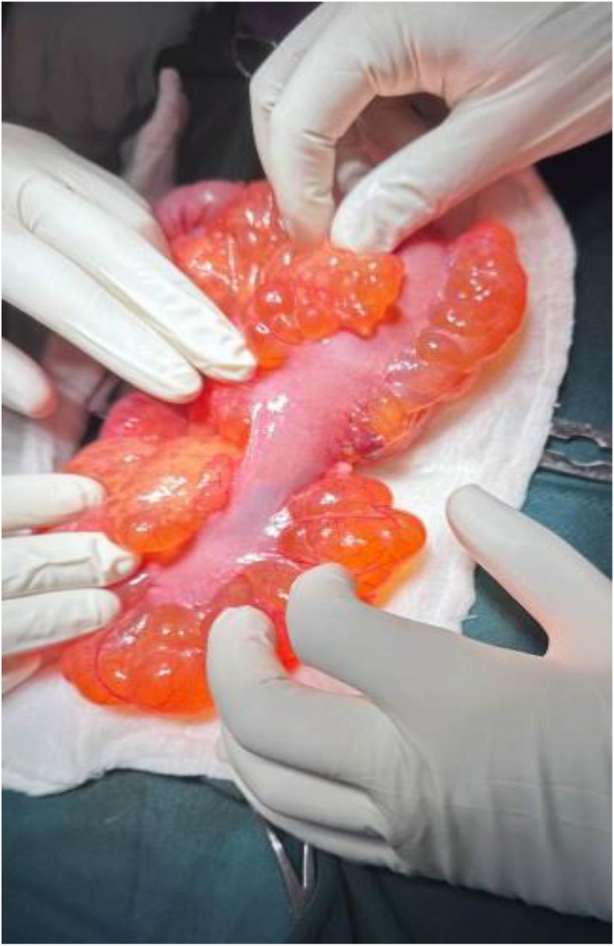
Intra-operative image demonstrating the ileum traversing through the mass with near circumferential encroachment.

**Figure 4.**
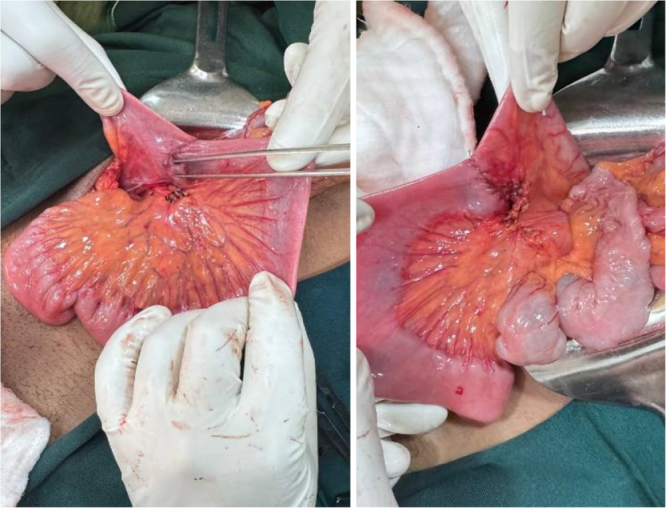
Intraoperative images showing the excision site following ileoileal end-to-end anastomosis.

**Figure 5.**
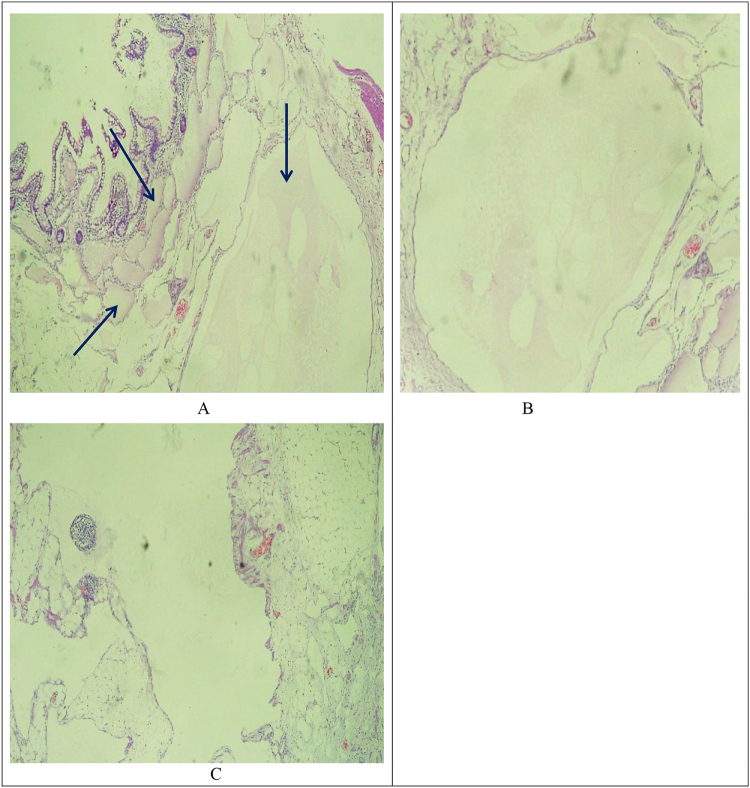
Light microscopy showing dilated and anastomosing vascular channels in the submucosa of the small intestine (A) and mesentery (B and C). The vascular channels are lined by flat endothelial cells and filled with proteinaceous material, likely a lymph fluid (pale eosinophilic material indicated with an arrow) (H&E stain).

Informed consent was taken, and the patient received prophylactic IV ceftriaxone (1 g, stat) and underwent exploratory laparotomy. Intraoperative findings revealed a smooth cystic mass lesion measuring about 15 × 10 × 6 cm at the distal ileum with near circumferential encroaching of 20 cm of ileum, starting 15 cm proximal to the ileocecal junction (Figs 2 and 3). The mass was excised *en bloc* with the involved bowel having a negative margin of about 5 cm, and an ileoileal end-to-end primary anastomosis was done (Fig. 4). Postoperatively patient was on IV Tramadol 50 mg TID, IM Diclofenac 50 mg BID, and daily wound care. Postoperative recovery was uneventful, and the patient was discharged on day 5. Histopathology confirmed mesenteric cystic lymphangioma (Fig. 5A–C).

On his 10th postoperative day, he was examined at the surgical referral clinic, and he was able to tolerate oral feeds, with no abdominal pain, distension, fever, vomiting or constipation. Further outpatient follow-up was done at 4 weeks and 6 months, following the surgery, the patient remained stable, and he had resumed normal activities.

## Discussion

Mesenteric cystic lymphangiomas are rare congenital tumors of lymphatic origin^[6]^. The mesenteric cyst was first described by an anatomist in Florence in 1507. Officially, the first description of mesenteric cystic lymphatic malformation was made by Rockitanski, and the first successful excision was performed by Tillaux in 1880^[3]^. The etiology is unclear, yet there are different theories^[7]^. It may be due to defective lymphatic system development, as failure of communication of lymphatic channels with the venous system results in sequestration and cyst formation. Another theory is obstruction of lymphatic channels due to trauma, hemorrhage, inflammation, surgery, and/or radiation therapy^[1,2,4,7]^. Lymphangiomas account for about 5–6% of all benign tumors in children, with a male predominance of 3:1 and a 1:1 ratio in adults^[1,6]^. Mesenteric cystic lymphangiomas are responsible for 1 in 20 000 child admissions and 1:100 000 adult admissions^[3]^. The most common sites of lymphangioma are the face and neck (60%), limbs (20%), thorax (10%), and axillary region (15%). Intra-abdominal lymphangiomas are very rare^[1]^. In the abdomen, lymphangiomas occur most commonly in the mesentery, followed by the omentum, mesocolon, and retroperitoneum^[4]^. Among intraperitoneal sites, 70–80% of cases occur in the small bowel mesentery, with 50–60% of these occurring in the ileal mesentery, and rarely in less than 1% of cases, the retroperitoneal space is involved^[1,3,7]^.

The clinical presentation of mesenteric lymphangioma is variable. They are usually asymptomatic and may be detected incidentally^[8]^. Patients may also present with an acute abdomen, including abdominal pain, distension, vomiting, fever, and abdominal tenderness. Less commonly, they may present with compression phenomena, hemorrhage, perforation, torsion, or rupture toward adjacent organs^[7]^. These cases are commonly large, and symptoms are likely due to the associated mass effect. Volvulus is not an uncommon presentation of small bowel mesenteric lymphangioma^[8]^. Differential diagnosis includes duplication cysts, hydatid disease, pancreatic cystadenoma, bowel adenocarcinoma, appendicular mucocele, tumor metastasis, cystic teratomas, and other rare mesenteric malignancies^[1,2]^.

Radiologic imaging is essential for preoperative diagnosis. A CT scan is considered the gold standard for providing detailed information on lesion density, adjacent organs, and distinguishing intraperitoneal from retroperitoneal lymphangiomas. Li *et al* reported a 90% diagnostic accuracy using abdominal ultrasonography and CT scan^[9]^. MRI is especially useful for evaluating cystic contents and perivascular extension, aiding surgical planning^[1,6,7]^. However, definitive diagnosis is always by histopathology^[6]^. In this case, a combination of contrast-enhanced abdominal CT and histopathology confirmed the diagnosis.

Lymphangiomas are classified histologically into capillary, cavernous, and cystic types, with the latter occurring intra-abdominally^[10,11]^. They are thin-walled cystic lesions lined by a single layer of endothelial cells, supported by lymphatic tissue and smooth muscle^[12]^. In this case, histology showed dilated vascular channels in the small intestine and mesentery filled with lymph fluid (Fig. 5A–C).

Although benign, intra-abdominal cystic lymphangiomas can cause bowel obstruction, perforation, or peritonitis and rarely transform malignantly. Complete surgical excision is the treatment of choice, often requiring bowel resection with anastomosis in over 50% of cases^[8,13,14]^. Limited negative margins are sufficient, and while laparoscopic resection is reported, open surgery is preferred for large masses due to lower recurrence^[7]^. Conservative management may be considered for asymptomatic lesions due to a 10% chance of spontaneous regression. Sclerotherapy and radiofrequency ablation have limited roles, and targeted therapies are reserved for aggressive or recurrent cases, but none surpass surgery in effectiveness^[1,4,6,7]^. Postoperative follow-up with clinical evaluation and ultrasound is recommended at 3, 6, and 12 months, then annually^[4]^. In this case, the patient remained stable with no recurrence.

This case highlights several important lessons; Adult mesenteric cystic lymphangiomas, though rare, should be considered in patients with chronic abdominal pain, discomfort, or palpable mass^[6,8]^. Radiologic imaging, particularly CT and MRI, is vital for surgical planning, but histopathology confirms the diagnosis^[1,6,7,12]^. Complete surgical excision with negative margins remains the gold standard, and recurrence is unlikely after total removal^[8,13,14]^. Early recognition prevents complications such as obstruction, perforation, infection, or rare malignant transformation^[1,6,13]^. Surgeons should carefully assess lesion size, location, and bowel involvement, and consider differential diagnoses before surgery to ensure optimal outcomes^[1,2,7]^.

Postoperatively, patients are usually followed with clinical examination and an abdominal ultrasound at 3, 6, and 12 months after the operation. Then, subsequent follow-up is recommended annually^[4]^. Currently, our case is on his routine postoperative follow-up.

The main limitation of this study is the inability to establish a cause–effect relationship or generalize the findings; however, it highlights an extremely rare clinical condition and provides high educational value.

## Conclusion

Mesenteric cystic lymphangiomas are exceedingly rare in adults. Diagnosis is challenging preoperatively due to non-specific symptoms. Mostly patients present with vague abdominal pain, discomfort and/or swelling. If complications occurred, patients may present with symptoms and signs of intestinal obstruction and/or perforation. Radiologic imaging, particularly CT and MRI, plays a crucial role in preoperative diagnosis, but histopathology confirms the diagnosis. Complete surgical excision is the best treatment modality. Recurrence is unlikely after complete excision with a negative margin.

## Data Availability

The authors of this manuscript are willing to provide any additional information regarding the case report.
